# To evaluate the performance of simultaneous amplification and testing assay for group B Streptococcus detection: comparison with real-time PCR and ddPCR assays

**DOI:** 10.1186/s12941-024-00726-y

**Published:** 2024-07-18

**Authors:** Loukaiyi Lu, Yisheng Chen, Qiang Wang, Jing Gao, Chunmei Ying

**Affiliations:** Department of Laboratory Medicine, Obstetrics and Gynecology Hospital of Fudan University, No 419 Fangxie Road, Shanghai, 200011 China

**Keywords:** Simultaneous amplification and testing (SAT), Real-time PCR (RT-PCR), Droplet digital PCR (dd PCR), Group B Streptococcus (GBS)

## Abstract

**Background:**

To evaluate the performance of simultaneous amplification and testing (SAT) assay for the detection of group B Streptococcus (GBS) in maternal vaginal and perianal swabs compared with real-time polymerase chain reaction (RT-PCR).

**Methods:**

We obtained vaginal and perianal swabs from 1474 pregnant women at the Obstetrics and Gynecology Hospital of Fudan University (Shanghai, China) between April 2023 and June 2023. Vaginal and perianal swabs were collected at 35–37 weeks of gestation. Swabs were tested for GBS simultaneously by using the SAT assay and RT-PCR, and a comparative analysis (kappa coefficient) was performed. Furthermore, we conducted additional droplet digital PCR (ddPCR) tests to confirm the results when there were controversial results between SAT and RT-PCR. In addition, we compared the limit of detection, technical specificity, repeatability and reproducibility of SAT-GBS with those of routine RT-PCR assays.

**Results:**

In our study, the detection rate of clinical GBS according to the SAT assay was 11.5% (169/1471). The SAT assay showed a sensitivity of 91.8%, a specificity of 99.9%, a diagnostic accuracy of 98.9%, a positive predictive value (PPV) of 99.4% and a negative predictive value (NPV) of 98.8%. The kappa value between RT-PCR and SAT was 0.917.

**Conclusions:**

This SAT assay for the detection of group B Streptococcus is not only easy to perform but can also detect GBS sensitively and specifically and may be used in the regular molecular diagnosis of GBS infection among pregnancies.

## Background

*Streptococcus agalactiae*, or group B Streptococcus (GBS), is widely acknowledged as a highly infectious bacterium that is associated with severe sepsis and meningitis among neonates and may cause neonatal morbidity and mortality [1]. According to previous studies, up to 30% of pregnant women may be infected by GBS [2] in the reproductive tract or the lower end of the digestive tract, and vertical transmission from mothers to newborns is strongly related to neonatal GBS colonization and even early-onset GBS sepsis [3]. Prevention is mainly based on intrapartum antibiotic prophylaxis (IAP) for GBS carriers, and thus, routine GBS screening in late pregnancy (35–37 weeks of gestation) is highly important [4]. A rational combination of IAP and prenatal GBS screening can effectively reduce the incidence of early-onset disease (EOD) [5].

The enrichment culture method is considered the gold standard method and is highly specific and accurate [6]; however, it is more time-consuming and less sensitive than molecular assays [7]. In the past few decades, molecular assays have been used in hospitals to speed up the diagnostic process so that timely clinical treatment can be provided [7]. To meet the demand of rapid diagnosis during late pregnancy or intrapartum detection in the labor ward, we thus call for an easier and faster detection method. The SAT assay requires only a single temperature and is less expensive than the RT-PCR assay, which often requires particular equipment to ensure rapid heating/cooling cycles [8]. Therefore, our study compared the performance of the SAT assay and RT-PCR in detecting GBS through clinical swabs before/during delivery in a Chinese hospital. Since ddPCR has been utilized in quantifying nucleic acid and detecting pathogens [9, 10] and previous study claimed that the limit of ddPCR for detecting GBS DNA was able to reach 5pg/µL [11], we used the ddPCR to confirm the controversial experimental results in our study.

## Materials and methods

### Clinical samples

We obtained swabs from 1474 pregnant women during late pregnancy (35 − 37 weeks of gestation) at the Obstetrics and Gynecology Hospital of Fudan University (Shanghai, China) from April to June 2023, and the study protocol was approved by the ethics committee of the Obstetrics and Gynecology Hospital of Fudan University (2023-81-X1). Sterile physiological saline was added to the specimens to elute bacterial cells from the swabs within 12 h of collection, and the eluates were divided equally into 3 tubes (Streck, USA). The first and second tubes of eluates were screened for GBS by using RT-PCR and SAT assays, respectively, and the results were recorded. In addition, the last tube was kept at -80 °C for further use.

### SAT assay for GBS

Simultaneous amplification and testing (SAT) is a nucleic acid detection method based on RNA transcription-mediated amplification and real-time testing using a molecular beacon probe [12]. The cfb gene of GBS has been used in the detection of GBS in multiple studies, and it has been shown to be highly conserved and suitable for GBS detection [13, 14]. Therefore, the cfb mRNA of GBS was reverse transcribed using Moloney murine leukemia virus (M-MLV) reverse transcriptase to generate a 150 bp DNA fragment with a T7 promoter sequence via specific primers, one containing the T7 promoter sequence and the other lacking it. Subsequently, T7 RNA polymerase recognizes the T7 promoter sequence and performs transcriptional amplification of RNA. The specific RNA beacon probe can hybridize with complementary sequences in the RNA amplicon and emit fluorescence signals. An internal control was included in the SAT assay. In this study, the experiments were conducted using reagents and protocols from Rendu Biotechnology. Briefly, 200 µL samples were mixed with 200 µL sample preservation solution provided by Rendu Biotechnology and heated at 95 °C for 10 min, followed by extraction using magnetic beads. All the extracted nucleic acids were added to the amplification system. The amplification and testing program was run at 42 °C for 40 min, and the fluorescence was measured every minute. The detection time (dt) refers to the minimum time (in minutes) when the fluorescence value reaches the threshold level. Specimens with FAM channel (GBS target) dt values ≤ 35 were considered GBS-positive. Specimens with FAM channel dt values > 35 and VIC channel (internal control) dt values ≤ 30 were classified as GBS-negative, while VIC dt values > 30 were deemed invalid. The extraction, amplification, and detection processes were carried out automatically in an automatic nucleic acid detection and analysis system (AutoSAT) manufactured by Rendu Biotechnology.

### Technical limit of detection, specificity, and repeatability of the SAT-GBS

*Streptococcus agalactiae* strains of different serotypes [serotypes Ia, Streptococcus agalactiae Lehmann and Neumann (ATCC-BAA-1138) and serotypes III, Streptococcus agalactiae Lehmann and Neumann (ATCC-BAA-2674) were purchased from American Type Culture Collection (ATCC)]. GBS strains were cultured in brain-heart infusion medium at 37 °C. The colony number was determined by the flat colony counting method. The initial culture concentrations were 4.35 × 10^7^ colony-forming unit/mL (CFU/mL), 3.95 × 10^7^ CFU/mL, 5.65 × 10^7^ CFU/mL and 2 × 10^8^ CFU/mL (serotypes Ia, Ib, III and V, respectively). The technical limit of detection (LoD) of SAT-GBS was evaluated by testing serial dilutions of the initial culture and reporting the 95% LoD through probability analysis. The endpoint LoD of the SAT-GBS and RT-PCR assays for GBS was estimated using 10-fold serial dilutions of the GBS culture sample (serotype III).

The specificity of the SAT-GBS was evaluated by testing microorganisms commonly present in the vaginal/rectal tract or related to the GBS family. The organisms were tested both with and without the GBS analyte at a concentration of 50 CFU/mL.

To evaluate the repeatability and reproducibility of SAT-GBS, two different serotypes of GBS culture (Ia and III) were tested at a concentration of 1 × 10^6^ CFU/mL. Each serotype was tested 10 times by the same operator in the same laboratory but 10 times by another operator in another laboratory on different days for the assessment of repeatability and reproducibility.

### Droplet digital PCR (ddPCR) assay for GBS

When we discovered controversial experimental results between SAT and RT-PCR for detecting GBS, we performed an extra ddPCR experiment to confirm whether the specimen was GBS positive. A rapid bacterial genomic DNA isolation kit (Sangon Biotech, China) was used to extract DNA from the last tube of eluates after they were incubated at room temperature for 30 min. ddPCR was performed in a QX200™ Droplet Digital PCR System (Bio-Rad Laboratories, CA) according to the manufacturer’s instructions [15]. A 20 µl volume of reaction mixture was required for each test, which comprised 10 µL of ddPCR Supermix for Probes (no dUTP; Bio-Rad), 1 µL of probe, 1 µL of forward primer, 1 µL of reverse primer and 5 µL of DNA template. For microdroplet generation, 20 µL of mixture and 70 µL of droplet generation oil were added to the DG8™ cartridge and then loaded into a QX200™ Droplet Generator. After that, 40 µL of the microdroplets were transferred to a 96-well PCR plate and heat-sealed with foil in the case of air pollution. Then, PCR was performed on a Bio-Rad T100™ PCR Thermal Cycler using the following conditions: predenaturation for 1 cycle at 95 °C for 3 min; denaturation for 40 cycles at 95 °C for 30 s; and annealing and extension for 40 cycles at 59 °C for 1 min (with a ramp rate of 2.5 °C/s). Finally, the fluorescence signal in each plate was analyzed by a QX200™ Droplet Reader and QuantaSoft™ Version 1.7.4, and each reaction used a negative control [11]. The threshold can be manually set according to the results of the negative control. °C.

In our study, specimens with quantification results > 1 copy/µL were defined as “true positive”, and specimens with quantification results < 0.5 copies/µL or lacking data were defined as “true negative”. When specimens had quantification results ranging from 0.5 copies/µL to 1 copy/µL (including 0.5 copies/µL and 1 copy/µL), the results were considered invalid, and these results were excluded.

### Primers and probes

For the RT-PCR assay, the primers and probes for the target and internal control sequences were supplied in the BioChain Strep B assay kit.

**Fig. 1:**
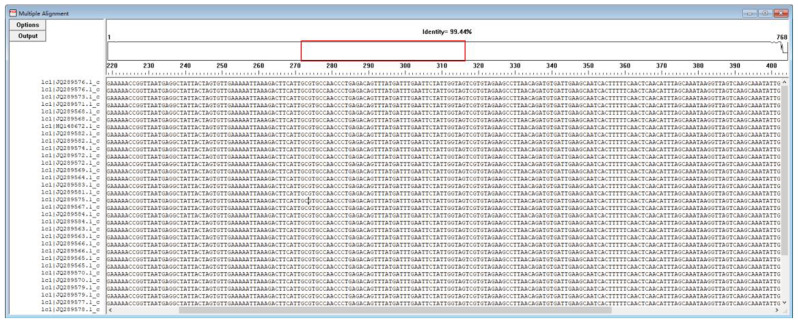
Comparison of the nucleotide sequences of genomes on the GBS region examined for the SAT-GBS primers and probe

For the SAT assay, primers and probes were designed to be specific for the mRNA of the GBS cfb gene. The cfb gene of GBS has been used in the detection of GBS in multiple studies, and it has been shown to be highly conserved [16]. The cfb sequence of the GBS strain (GenBank Genomic Sequence: NZ_CP012480.1, 1929499 to 1930266) was obtained from the GenBank database and used in the design of primers and probes for SAT-GBS via DNAMAN software (Fig. 1). The forward primer contained the T7 promoter sequence (Table 1). The probe was labeled with FAM at the 5’ end and with the quencher DABCYL at the 3’ end. The IC probe sequence was labeled with HEX at the 5’ end and with DABCYL at the 3’ end.

**Table 1:** Prime, probe and IC-RNA sequences of SAT assay

Forward primer	5’*AATTTAATACGACTCACTATAGGGAGA*GTTAAGGCTTCTACACGACT−3’
Reverse primer	5’AATTTAATACGACTCACTATAGGGAGAGTTAAGGCTTCTACACGACT−3’
Probe	5’GAGACAGUUUAUGAUUUGUCUC−3’
IC-probe	5’-CCGACGUGAUACGAGAGAGUCGG−3’

The sequence marked with an underline is T7 promoter sequence

For the ddPCR assay, we selected the tyrosine protein kinase (TPK) gene as the target gene according to previous studies [17]. The sequence of *Streptococcus agalactiae* TPK was obtained from the NCBI database and used in the design of primers and probes for ddPCR-GBS (Table 2).

**Table 2:** Prime and probe sequences of ddPCR assay

Primer	Sequence
Forward primer	CGCCGTAAGTAGCAACAGAT
Reverse primer	AAAGAACAGATGGAACAAAGT
Probe	AGAATAATACCTAAGAACTTTGAACC

### Statistics

Statistical data analyses were performed by SPSS Statistics Version 24.0. The specificity, sensitivity, NPV and PPV were calculated with a 2 × 2 contingency table, and chi-square tests/Fisher’s exact tests and kappa tests were carried out where appropriate. A *p* value < 0.05 was considered to indicate statistical significance.

## Results

### Limit of detection of the SAT-GBS

**Fig. 2:**
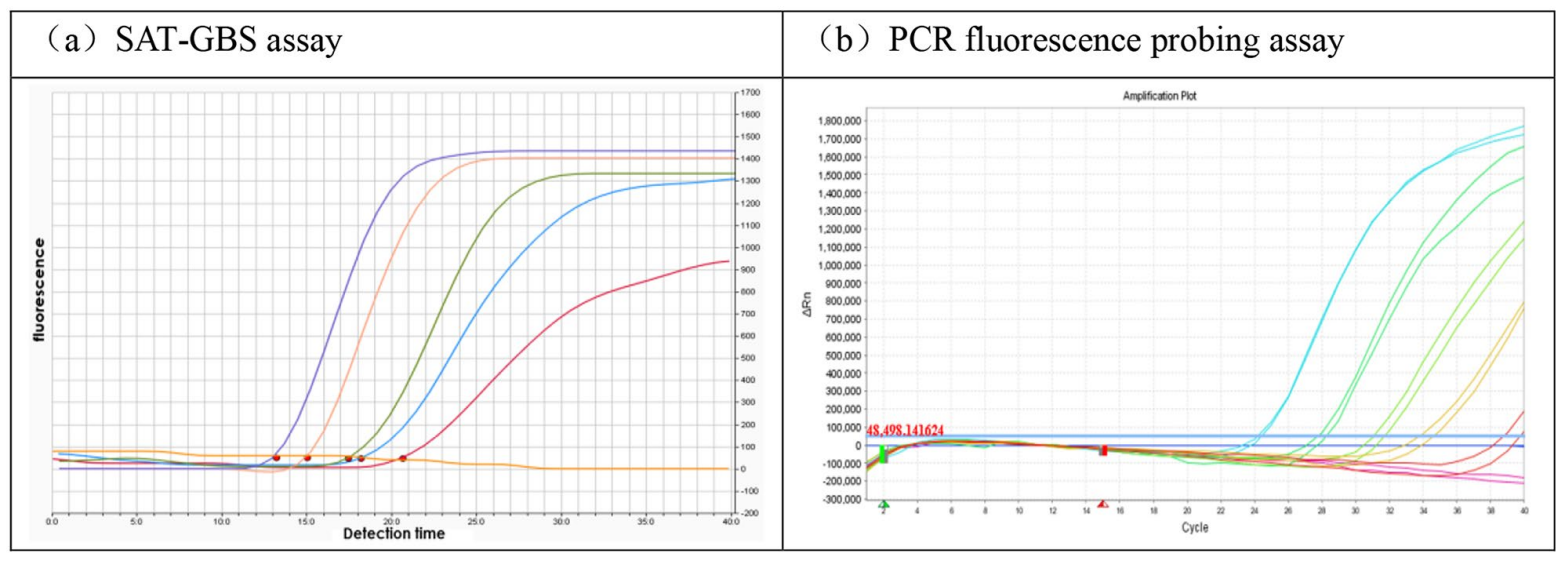
Comparison of the sensitivity between the SAT-GBS assay and PCR-fluorescence probing assay. Amplification curves of SAT-GBS assay (**a**) and PCR fluorescence probing assay (**b**). The concentrations of the samples in the figure from left to right are 2.5 × 10^5^ CFU/mL、2.5 × 10^4^ CFU/mL、2.5 × 10^3^ CFU/mL、2.5 × 10^2^ CFU/mL、25 CFU/mL. Based on the test results, SAT-GBS assay has a more easily distinguishable detection result than PCR-fluorescence probing assay

By using 10-fold serial dilutions of the GBS culture sample, the technical limit of detection of SAT-GBS was assessed. When the optimal amount of IC was used (5 × 10^6^ copies per reaction), SAT-GBS was found to successfully amplify from 2.5 × 10^5^ CFU/mL to 2.5 × 10 CFU/mL. To compare the LoD of the two assays, the SAT-GBS and RT-PCR assays were assessed down to 2.5 × 10 CFU/mL (Fig. 2). In our study, we found that the SAT-GBS assay was more sensitive than the RT-PCR assay for detecting GBS.

### Specificity of the SAT-GBS

To evaluate the technical specificity of the SAT-GBS, we tested a panel of microorganisms, including viruses, bacteria, fungi and protozoans. Moreover, bacteria were tested at 1 × 10^6^ CFU/mL, viruses and fungi were tested at 1 × 10^5^ copies/mL, and protozoans were tested at 1 × 10^5^ cells/mL. Luckily, none of the pathogens were found to have cross-reacted in the SAT-GBS.

### Repeatability and reproducibility of the SAT-GBS

The repeatability and reproducibility of SAT-GBS were assessed through calculation of the coefficient of variation (CV) in the detection time (dt) of positive amplification plots, in which dt values were regressed to predict the amount of target RNA. In a single run of the two different serotypes at a concentration of 1 × 10^6^ CFU/mL, the CVs were 2.81% and 3.27%, respectively. In different runs, the CVs of the two different serotypes were 4.01% and 4.00%, respectively (Table 3).

**Table 3:** Coefficient of variation (CV) of serotype Ia and III

	RUN 1		RUN 2	
Serotype	Ia	III	Ia	III
TEST 1	15.4	15	15.2	13.9
TEST 2	15.2	14.1	14.4	13.9
TEST 3	15.4	14.5	14.5	13.8
TEST 4	16.3	14.7	15.3	13.9
TEST 5	16	15.1	15.2	13.2
TEST 6	16.1	15.2	15	14.1
TEST 7	15.7	15.3	14.3	13.9
TEST 8	14.9	14.6	14.2	13.6
TEST 9	16	14.5	15.3	14.2
TEST 10	15.7	13.8	14.8	14.4
CV of Ia and III in RUN 1	2.8%	3.3%	/	/
CV of Ia and III BETWEEN RUNS	4.0%	4.0%	4.0%	4.0%

### SAT-GBS in clinical specimens

The sensitivity and specificity of the SAT-GBS were also evaluated by using 1474 clinical maternal vaginal and perianal swabs. By comparing SAT-GBS with RT-PCR, we discovered 1448 concordant results and 26 discordant results. Among these discordant results, 20 were negative according to the SAT-GBS assay but positive according to the RT-PCR assay, and after reconfirmation by the ddPCR assay, 15 were confirmed to be true positive, and these 15 “SAT-GBS assay negative” results were considered false negatives. In addition, 3 of the 20 controversial results were defined as invalid results, were excluded because of relatively low ddPCR quantification results and were considered unreliable. Furthermore, one of the remaining six discordant results was identified as a false positive since the SAT assay was positive, while the RT-PCR and ddPCR assays were negative (Table 4).

**Table 4:** The results of the 26 discordant specimens by RT-PCR assay, SAT assay and ddPCR assay

No.	SAT Assay (dt value)	PCR Assay (Ct value)	ddPCR Assay (copies/µL)	Result
1	0	36.75	0	N
2	19.3	0	1.5	P
3	0	36.76	1.4	P
4	0	35.8	9.7	P
5	0	37.68	1	un
6	0	33.25	1.2	P
7	0	37.39	2.3	P
8	23.9	0	8.6	P
9	0	38.02	3.7	P
10	0	38.37	2.8	P
11	0	35.83	3.1	P
12	19.6	0	11.2	P
13	0	31.21	40.0	P
14	0	36.21	1.5	P
15	19.9	0	0.47 < 0.5	N
16	0	37.09	3.3	P
17	0	37.15	1.8	P
18	0	36.98	2.2	P
19	0	36.09	4.3	P
20	0	37.12	12.4	P
21	0	37.47	0	N
22	15.9	0	2.3	P
23	18.4	0	20.1	P
24	0	36.75	2.5	P
25	0	37.99	0.74	un
26	0	36.15	0.85	un

After the exclusion of the 3 invalid results, the SAT assay showed a sensitivity of 91.8%, specificity of 99.9% and diagnostic accuracy of 98.9%. The PPV was 99.4%, and the NPV was 98.8% (Table 5). The kappa value between RT-PCR and SAT was 0.917.

**Table 5:** Diagnostic performance of RT-PCR assay and SAT assay in clinical specimens

Methods	Turnaround Time	TP	TN	FP	FN	NPV	PPV	Sensitivity (95% CI)	Specificity (95% CI)	Diagnostic Accuracy
RT-PCR	4 h	182	1287	2	3	99.6%	98.9%	97.3% (93.4–99.0)	99.8% (99.4–100.0)	99.7%
SAT	2 h	170	1288	1	15	98.8%	99.4%	91.8% (86.6–95.2)	99.9% (99.5–100.0)	98.9%

*TP, true positive; TN, true negative; FP, false positive; FN, false negative; CI, confidence interval; PPV, positive predictive value; NPV, negative predictive value; Sensitivity = [TP/(TP + FN)] × 100%; Specificity = [TN/(TN + FP)] × 100%; PPV = [TP/(TP + FP)] × 100%; NPV = [TN/(TN + FN)] × 100%

## Discussion

In our study, the SAT-GBS revealed a GBS detection rate of 11.5% (169/1471), which was close to that reported in previous studies in mainland China (14.5%), Korea (11.6%) and Japan (18.2%) [18–21] but lower than that reported in America (21.6%) [22] and Hong Kong (21.8%) [23], probably due to various factors, including ethnicity, health conditions, social economy, sampling and screening methods, etc. [19]. In addition, the detection rates determined by the SAT and RT-PCR showed no significant differences according to the chi-square test (*p* = 0.531, data not shown). Despite the fact that enrichment culture has been considered the gold standard for GBS screening in the USA [24] and Europe [25], direct blood agar plate culture has been widely performed in China to save time and reduce costs [19]; thus, the false negative rates in Chinese laboratories are high according to previous studies [26]. During our preexperiment period (comprising 269 specimens), we compared the SAT assay with a direct culture assay (the swab was collected on blood agar before elution). The preexperiment showed a relatively low culture assay positive rate of 7.06% (19/269), which is similar to that reported in Korea (4.4%), and Korean studies have shown that the rate of GBS colonization determined by culture assay varies depending on the institution, even within the same country [27]. In our hospital, RT-PCR has been applied in routine prenatal examinations for GBS screening since September 2013 in patients’ late pregnancy. Considering these factors, this diagnostic accuracy study focused on comparisons between RT-PCR assays and SAT assays, unlike most other studies in which enrichment culture was used as the reference method [8, 19]. In addition, in our study, when we found controversial results between the SAT assay and RT-PCR assay, an extra ddPCR experiment was conducted to confirm the results, which was believed to be able to provide accurate quantification of pathogen nucleic acid [10]. The reason why we defined the 3 RT-PCR assay results as weakly positive (the FAM Ct values of the 3 results were 37.68, 37.99 and 36.15) with negative SAT assay results as “invalid” was that the reexamined ddPCR results were 1 copy/µL, 0.74 copies/µL and 0.85 copies/µL, respectively, and we could not tell whether the 3 samples were negative or positive. Sample-related uninterpretable factors, including inadequate sampling and DNA-degrading substances, may explain the appearance of invalid results.

In this study, we found that the SAT assay has relatively good sensitivity, specificity, PPV and NPV. Moreover, since our SAT assay requires only a single temperature for amplification, the turnaround time is superior to that of existing RT-PCR assays and enrichment cultures [8]. SAT technology only requires 40 min of amplification time to achieve ideal results, while PCR amplification time is generally around one and a half hours. Due to the high expression of RNA only in live bacteria, RNA detection results can be indirectly used for therapeutic evaluation. Previous studies suggested that GBS screening results should be provided at least 4 h before the birth process so that targeted intrapartum prophylaxis can be conducted [4], and our SAT-GBS assay may meet this demand if a 24-hour operating diagnostic laboratory is available.

A limitation of our study was that our samples were collected from vaginal and perianal regions, while many studies revealed that rectal colonization was more common than vaginal colonization [28], which means that we may have failed to detect some rectally colonized GBS-positive patients, and our GBS detection rate was relatively lower than the real situation, which may also explain why our GBS-positive rate was much lower than that in the USA [22].

Another limitation of our study was that our GBS-SAT assay lacks antimicrobial susceptibility testing, which means that even with the 24-hour availability of GBS screening results, doctors can only treat patients with empirical therapy at the time of membrane rupture or in the labor process. Considering the the possibility of betalactam allergy, *Penicillin* skin testis required.

It has been proven that the colonization status of GBS may change during pregnancy. However, early study has already demonstrated that cultures taken less than 5 weeks before delivery can accurately predict the GBS carrier status at delivery [29]. In addition, there are still many pregnant women who deliver their babies without GBS prenatal screening tests due to preterm birth [27]. Overall, our SAT-GBS assay may reach the demand of finding an accurate and rapid method for late antenatal and intrapartum GBS screening.

## Conclusions

The simultaneous amplification and testing assay performed well in detecting GBS, which may satisfy the clinical demand for developing a highly accurate and rapid GBS screening method and reduce the incidence of newborn sepsis and meningitis.

## Data Availability

No datasets were generated or analysed during the current study.

## Abbreviations

SAT: Simultaneous amplification and testing
GBS: Group B Streptococcus
RT‒PCR: Real-time polymerase chain reaction
ddPCR: Droplet digital PCR
PPV: Positive predictive value
NPV: Negative predictive value
IAP: Intrapartum antibiotic prophylaxis
EOD: Early-onset disease
M-MLV: Moloney murine leukemia virus
Dt: Detection time
ATCC: American Type Culture Collection
CV: Coefficient of variation
CFU: Colony-forming unit
TPK: Tyrosine protein kinase
LoD: Limit of detection
